# Significant Low Prevalence of Antibodies Reacting with Simian Virus 40 Mimotopes in Serum Samples from Patients Affected by Inflammatory Neurologic Diseases, Including Multiple Sclerosis

**DOI:** 10.1371/journal.pone.0110923

**Published:** 2014-11-03

**Authors:** Elisa Mazzoni, Silvia Pietrobon, Irene Masini, John Charles Rotondo, Mauro Gentile, Enrico Fainardi, Ilaria Casetta, Massimiliano Castellazzi, Enrico Granieri, Maria Luisa Caniati, Maria Rosaria Tola, Giovanni Guerra, Fernanda Martini, Mauro Tognon

**Affiliations:** 1 Department of Morphology, Surgery and Experimental Medicine, Section of Pathology, Oncology and Experimental Biology, University of Ferrara, Ferrara, Italy; 2 Unit of Neuroradiology, University Hospital of Ferrara, Ferrara, Italy; 3 Biomedical Sciences and Specialized Surgeries, Section of Neurology, School of Medicine, University of Ferrara, Ferrara, Italy; 4 Unit of Neurology, University Hospital of Ferrara, Ferrara, Italy; 5 Clinical Laboratory Analysis, University Hospital of Ferrara, Ferrara, Italy; National Institutes of Health, United States of America

## Abstract

Many investigations were carried out on the association between viruses and multiple sclerosis (MS). Indeed, early studies reported the detections of neurotropic virus footprints in the CNS of patients with MS. In this study, sera from patients affected by MS, other inflammatory (OIND) and non-inflammatory neurologic diseases (NIND) were analyzed for antibodies against the polyomavirus, Simian Virus 40 (SV40). An indirect enzyme-linked immunosorbent assay (ELISA), with two synthetic peptides, which mimic SV40 antigens, was employed to detect specific antibodies in sera from patients affected by MS, OIND, NIND and healthy subjects (HS). Immunologic data indicate that in sera from MS patients antibodies against SV40 mimotopes are detectable with a low prevalence, 6%, whereas in HS of the same mean age, 40 yrs, the prevalence was 22%. The difference is statistically significant (*P = *0.001). Significant is also the difference between MS vs. NIND patients (6% vs. 17%; *P* = 0.0254), whereas no significant difference was detected between MS vs OIND (6% vs 10%; *P*>0.05). The prevalence of SV40 antibodies in MS patients is 70% lower than that revealed in HS.

## Introduction

Multiple sclerosis (MS) is a chronic human demyelinating disease of the central nervous system (CNS) characterized by an autoimmune pathogenic process in genetically predisposed individuals [1], [2]. Although the etiology of MS is unknown, genetic and environmental factors seem to play an important role.

Accumulating data, including animal study models, human models of virus inducing demyelination, epidemiologic and laboratory findings, have demonstrated that viruses and host genetic factors can interact to cause immune-mediated demyelination [3], [4]. Infectious agents are environmental factors potentially involved in the MS onset. Specifically, ubiquitous viruses were found associated with the development or exacerbation of MS, including the *herpesviruses* (i) *Epstein-Barr virus* (EBV) [5], [6], [7], [8], (ii) *human herpesvirus 6* (HHV-6) [9] and *human endogenous retrovirus* (HERV) families [10]. Although the current evidence supports a strong association between EBV and MS, the potential causality of this herpesvirus remains to be established [11].

EBV has been investigated for its putative role in the MS onset. Earlier studies found a higher prevalence of anti-EBV antibodies in MS patients compared to controls [7], [8]. At present, it cannot be excluded that the abnormal response to EBV infection in MS patients is a consequence, rather than a cause. It has been reported that EBV cannot alone trigger the MS onset [7]. Further molecular evidences are needed to assess the real involvement of EBV in the MS onset.

HHV-6 strains A/B has been proposed as viral agents involved in several autoimmune disorders (AD), including MS. HHV-6A could participate in neuro-inflammation in the context of MS by promoting inflammatory processes through CD46 binding [9].

HERV-Fc1, which sequences map in chromosome X, has been associated with MS, mostly in Northern European populations. Association of the HERV-Fc1 polymorphism rs391745 with bout-onset MS susceptibility was also confirmed in Southern European cohorts [10].

Polyomaviruses, including Simian Virus 40 (SV40) [12], [13] have been poorly investigated for their putative role in MS disease [14]. SV40, a monkey neurotropic polyomavirus, is responsible for the progressive multifocal leukoencephalopathy (PML) in immune-compromised macaques [15], [16] while in humans its footprints have been detected in brain tumors and neurologic disorders [17], [18], [19].

Recently, the development of specific and sensitive serologic test for SV40 has been reported, which consists of an indirect ELISA employing synthetic peptides as mimotopes/antigens of SV40 viral capsid proteins (VPs). This immunologic assay was used to detect specific serum antibodies against SV40 VPs in normal individuals of different age [20], [21], [22]. Higher prevalence of SV40 antibodies was detected in oncologic patients affected by glioblastoma multiforme (GBM) [19], whereas SV40 sequences and large T antigen expression were detected in human brain tumors [23], [24], [25].

The complex interactions among the CNS, multiple infections with different infectious agents occurring in the periphery or within the CNS, and the immune response should be analyzed and elucidated in order to understand the etiology of MS.

The objective of the present study was to investigate whether serum samples from patients affected by MS, other OIND, NIND and HS carry SV40-antibodies. Sera were analyzed by an indirect ELISA employing synthetic peptides as mimotopes belonging to the viral capsid proteins (VPs).

## Results

### Low prevalence of antibodies reacting with SV40 mimotopes in serum samples from multiple sclerosis patients

Serum samples from MS patients were analyzed by indirect ELISA for the presence of IgG class antibodies against SV40 VP mimotopes/epitopes (Tables 1 and 2). The indirect ELISA was employed to test serum samples of MS affected patients (mean age  = 37 yrs), which had been diluted at 1/20, for reactivity to SV40 epitopes from VP1, VP1 B peptide. Serum samples reacting with the SV40 VP1 B mimotopes reached an overall prevalence of 9%. Then, the same assay was addressed to detect IgG class serum antibodies against SV40 VP2/3 epitopes, which are known as VP2/3 C peptide. Serum samples reacted with the SV40 VP2/3 C peptide with a similar prevalence, 13%, as had been detected previously for the VP1 B peptide. Conversely, seronegative samples for the SV40 VP1 B peptide failed to react with SV40 VP2/3 C epitopes. The exceptions were negligible represented by a few serum samples, which were negative for VP1 B peptide, while testing positive for VP2/3 C peptide, and vice-versa. The difference was not statistically significant (*P*>0.05) (Table 1). The different prevalence of responses/OD reading of B and C peptides is probably due to the different immunogenicity of the two SV40 antigens. Indeed, the B peptide is present in the VP1 virion, in its pentameric form, 360 times, whereas the C peptide of VP2/3 is present in the virion 72 times [20].

**Table 1:** MS, OIND and NIND patients and healthy subjects.

Serum Subject (N)	Female (%)	Mean yrs ± SD (range)	Subtype	Number Of Subtype	SV40-positive sample/sample analyzed (%)
**MS**	66	37±11.2	RR	78	6/78 (7)
(93)		(13–68)	SP	10	0/10
			PP	5	0/5
**OIND**	50	53±14.2	Inflammatory Demyelinating	34	2/34(6)
(77)		(18–83)	Meningitis/Encephalitis/Myelitis	25	3/25(12)
			Mononeuritis	4	0/4
			Comnnectivitis/Vasculitis	13	3/13(23)
			PML	1	0/1
**NIND**	50	52±16.8	ALS	8	2(25)
(81)		(21–85)	Dementia	6	0/6
			MSA	1	0/1
			Arteriovenous malformation	3	1/3(33)
			Migraine	10	5/10(50)
			Toxic encephalopathy	2	0/2
			Epilepsy	4	0/4
			Hereditary ataxia	3	1/3(33)
			Brain Tumor	8	0/8
			Hydrocephalus	2	0/2
			Spondylotic myelopathy	4	0/4
			Peripheral neuropathy	9	2/9(22)
			Pseudotumor cerebri	2	1/2(50)
			Funicular myelopathy	1	0/1
			Stroke/TIA	18	2/18(11)
**HS1**	67	40±11.7	Healthy subjects	180	40/180(22)
(180)		(17–67)			
**HS2**	50	51±11.7	Healthy subjects	160	34/160(21)
(160)		(18–81)			

MS (Multiple Sclerosis); OIND (Other Inflammatory Neurologic Disease); NIND (Non Inflammatory Neurologic Disease); HS (Healthy Subjects); RR (Relapsing-Remitting): SP (Secondary-Progressive); PP (Primary-Progressive); PML (Progressive Multifocal Leucoencephalopathy); ALS (Amyotrophic Lateral Sclerosis); MSA (Multiple System Atrophy); TIA (Transient Ischemic Attack). Among SV40-positive patients, 2 OIND patients were found to be affected by Inflammatory Demyelinating, 3 patients were affected by Meningitis/Ancephalitis/Myelitis and 3 by Comnnectivitis/Vasculitis. Among NIND patients SV40-positive were 2 patients affected by Amyotrophic Lateral Sclerosis (ALS), 1 by Arteriovenous Malformation, 1 by Hereditary Ataxia, 2 by Peripheral Neuropathy, 1 by Pseudotumor Cerebri and 2 by Transient Ischemic Attacks (TIA). The three MS patients were affected by relapsing remitting MS forms.

**Table 2:** Prevalence of immunoglobulin G antibodies reacting with Simian Virus 40 (SV40) viral protein (VP) mimotopes in serum samples of patients affected by MS, OIND, NIND and HS∧.

Serum∧ sample	Number of patients/subject	Female %	Number of positive samples (%)		
			VP B	VP C	VP B+C
**MS**	93	66	8 (9)	12 (13)	6 (6)*
**HS1**	180	67	63 (35)	52 (29)	40 (22)
**OIND**	77	50	20 (26)	11 (14)	8 (10)**
**NIND**	81	50	16 (20)	16 (20)	14 (17)
**HS2**	160	52	45 (28)	40 (25)	34 (21)

∧Human sera were from patients affected by multiple sclerosis (MS), other inflammatory neurologic diseases (OIND), non-inflammatory neurologic diseases (NIND) and healthy subjects (HS1), (HS2).

*The prevalence of SV40 antibodies in MS patients is statistically significant lower that those detected in NIND patients (*P* = 0.0254) and in HS1 (*P* = 0.001), whereas no significant was the different prevalence detected in MS and OIND patients (*P*>0.05).

** The prevalence of SV40 antibodies in OIND patients is statistically significant lower that those detected in HS2 (*P* = 0.0403). The different prevalence of SV40 antibodies between the cohorts of NIND patients was not significant compared with the HS2 (*P*>0.05). Statistical analysis was performed using the χ^2^ test.

As published before [19], [20], [21], [22] in indirect ELISAs the human peptide hNPS which is unrelated to SV40, was employed as a negative control peptide to verify if, in our experimental conditions, a non-specific reaction may occur with SV40-positive and SV40-negative human and rabbit serum samples. Data indicate that this negative control peptide does not react with SV40-positive and SV40-negative sera. The OD value was usually in the range of 0.088-0.098, which is consistent with the OD background of both human and rabbit sera [19], [20], [21], [22].

The two indirect ELISAs, with the two distinct VP B and C peptides gave overlapping results, thus confirming the presence of anti-SV40 VPs antibodies, although at low prevalence, in human sera from patients affected by MS (Tables 1 and 2).

In our investigation only those samples found positive for both B and C peptides were considered SV40-positive (Tables 1 and 2).

Altogether, our immunologic data indicate that combining the SV40-positive sera (6/93), both for the VP1 B and VP2/3 C peptides, the overall prevalence was 6% (Tables 2).

Control samples represented by sera from healthy subjects, with a similar mean age of MS patients, 40 yrs, were investigated with the same ELISA. Immunologic results indicated a prevalence 22% in these healthy subjects (HS1) (Table 2). These results are in agreement with previous immunologic data, obtained with other cohorts of HS, with the same mean age [20]. Indeed, ELISA data indicate that the prevalence of SV40 antibodies is similar in the cohorts of individuals with the same age and gender, despite coming from different regions in Italy. However, a decline of prevalence was detected in elderly [21].

**Figure 1:**
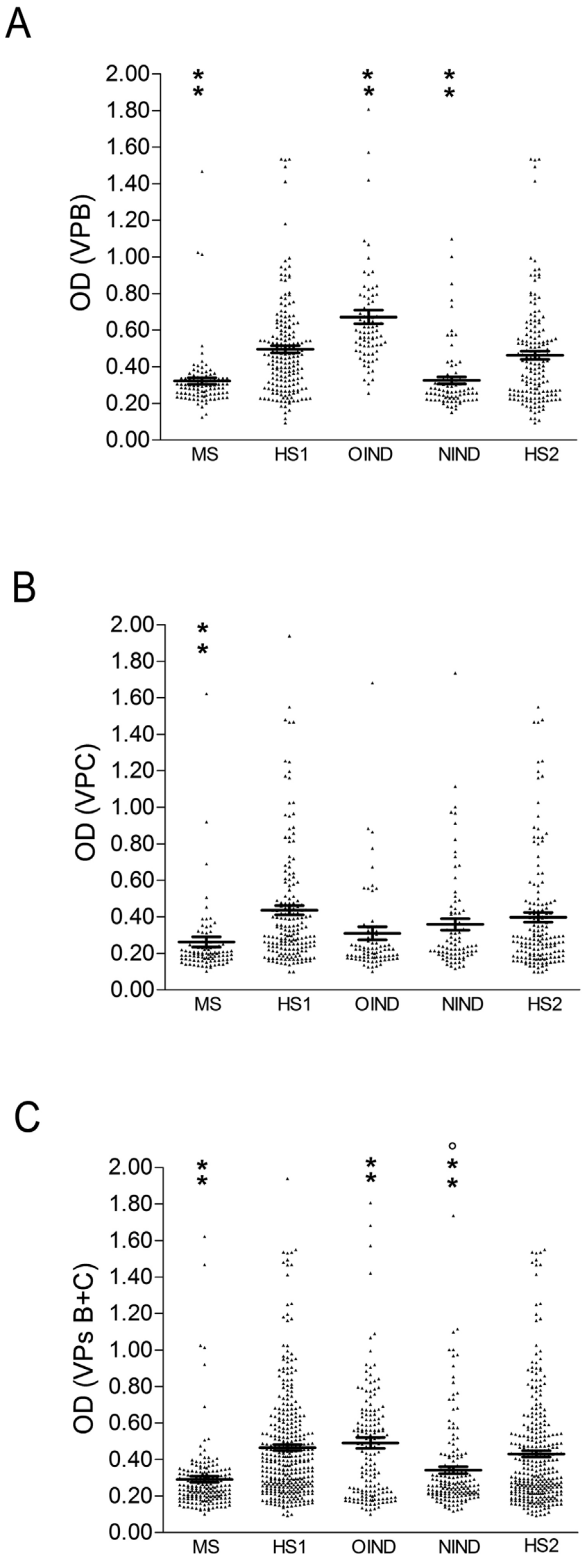
Serologic profile of serum antibody reactivity to SV40 mimotopes VP1 B (A) and VP2/3 C (B) and VPs B+C (C). Immunologic data are from serum samples from Multiple Sclerosis patients (MS), Healthy Subjects (HS1), Other Inflammatory Neurologic Diseases (OIND), Non-Inflammatory Neurologic Diseases (NIND) and Healthy subjects (HS2). Results are presented as values of optical density (OD) readings at λ 405 nm, of serum samples diluted at 1∶20, detected in indirect ELISA. In scatter dot plotting, each plot represents the dispersion of OD values to a mean level indicated by the line inside the scatter with Standard Error Mean (SEM) for each group of subjects analyzed. A) The mean OD of sera (VP B ± Std Error) in MS (0.32±0.02) were lower than that in HS1 (0.50±0.02) and in OIND (0.67±0.04). Moreover the mean OD of sera in OIND were higher than that in NIND (0.32±0.02) and HS2 (0.46±0.02). The mean OD of sera in NIND were lower than that in HS2. B) The mean OD of sera (VP C ± Std Error) in MS (0.26±0.03) were lower than that in HS1 (0.43±0.02). C) The mean OD of sera (VPs B+C ± Std Error) in MS (0.29±0.02) were lower than that in HS1 (0.46±0.02) and in OIND (0.49±0.03). Moreover, the mean OD of sera in OIND were higher than that in NIND (0.34±0.02). The mean OD in NIND were lower than that in HS2 (0.43±0.02) and in OIND sera. Statistical analysis was performed using Anova and Newman-Keuls Comparison test. (**P<0.001; °*P*<0.01).

MS and HS1 serologic profiles of serum antibody reactivity to SV40 mimitopes are shown in Figure 1.

SV40-positive sera, which were selected by the neutralization assay as reported elsewhere [19], [20] tested by indirect ELISA diluted at 1/20 had a general cut-off, by spectrophotometric reading, in the range of 0.18 OD. This cut-off represents in our ELISA the value that discriminates SV40-negative (OD <0.18) from SV40-positive samples (OD>0.18).

### ELISA data obtained with serum samples from OIND, NIND patients and HS

In the second step of this investigation, indirect ELISA was employed to test serum samples from OIND and NIND affected patients, and healthy subjects (HS2), with a similar mean age of these patients (51 yrs). Sera had been diluted at 1/20, for reactivity to SV40 epitopes from VP1, VP1 B peptide and then from VP 2/3, C peptide. Serum samples, which reacted with both SV40 VP1 B and VP2/3 mimotopes reached an overall prevalence of 10% in OIND, 17% in NIND, whereas the control samples represented by sera from HS2 the prevalence was 21%.

Serologic data obtained with the cohorts of HS1 and HS2 are in agreement with those reported before with other cohorts of HS, with the same mean age [20].

It is interesting to note that SV40 peptide C prevalence in these samples is very similar to the data obtained with the same samples for SV40 peptide B. OIND, NIND and HS2 serologic profiles of serum antibody reactivity to SV40 mimotopes are shown in Figure 1.

## Discussion

SV40-antibody detection had been attempted in several studies using serologic methods with SV40 antigens, but due to the high homology among the three main Polyomaviruses (SV40, BKV and JCV), the results were always affected by some cross-reactivity [14], [17], [26], [27], [28], [29].

Specific immunologic assays for the identification of SV40-seropositive individuals/patients and serum antibody reactivity to SV40 antigens are of paramount importance in revealing the prevalence of SV40 infection in patients and normal subjects.

In this study, serum samples from patients affected by neurologic diseases, including MS, together with those from healthy individuals employed as controls, were analyzed for exposure to SV40 infection. The indirect ELISA employed in this study used specific SV40 antigens/mimotopes. Indeed, the synthetic peptides from SV40 VP 1–3 employed mimic the corresponding epitopes of the viral capsid peptides [19], [20], [21], [22]. Our immunologic data suggest that specific SV40 antibodies are detectable in human serum samples from neurologic patients and healthy individuals.

The prevalence of SV40 antibodies observed in Italian patients affected by MS and OIND, revealed a lower prevalence of SV40 antibodies in these patients respect to controls represented by NIND patients and HS. Indeed, our serologic assays indicated that the prevalence of SV40 antibodies in serum samples of patients affected by MS (6%) is lower than that determined in HS1 (22%) (P = 0.001) and in NIND patients (17%) (P = 0.0254). Similarly, the prevalence of SV40 antibodies determined in OIND patients (10%) is lower than that determined in HS2 (21%) (P = 0.04).

It should be noted that MS patients included in our study, at the time of the serum collection, were not subjected to any immuno-modulatory therapy. This data indicates that the low prevalence of SV40 antibodies is not due to the immune-modulatory therapy.

It has been proposed that viruses may play a role in MS pathogenesis acting as environmental triggers. However, it remains to be elucidated whether viruses are causal agents of the MS onset.

Our data represent the first study indicating a significant low prevalence of serum antibodies against SV40 in patients affected by inflammatory neurologic diseases, including MS. One may speculate that these patients, with specific impairments of their immune system, are poor responders to SV40 infection because unable to present these polyomaviral antigens. In this peculiar condition of the host, SV40 escaping from the immune surveillance could exerts its pathologic effect in human oligodendrocytes. Indeed, SV40 is responsible in immunodepressed/suppressed macaques of the PML, another inflammatory neurologic disease related to the myelin degradation [15], [16]. It should be noted that SV40 in HIV-positive or AIDS patients does not increase its viral load indicating that human cells are only semi-permissive to its multiplication, whereas the inflammatory molecules do not efficiently reactivate this polyomavirus [30], [31], [32].

A recent investigation reported that the IgG levels in MS patients against distinct herpesviruses do not differ from that of controls, whereas a higher IgG prevalence against EBV was detected [5]. Our immunologic data suggest that a specific dysregulation in the IgG response to SV40 in multiple sclerosis and OIND patients may occur. At present, the nature of this immunologic defect is not known. It has been reported that the cronic inflammation, which characterize these patients, is responsible of several immune impairments [33]. The low level of SV40 antibodies detected in serum samples of MS patients could be related to the cronic inflammatory conditions of these patients that may affect the response/stability of SV40 antibody over time.

Alternatively, the low prevalence of SV40 antibodies detected in these patients indicates that they are less prone to the SV40 infection, or a closely related yet unknown human polyomavirus.

Additional studies are needed to verify if SV40 plays a role in the MS onset, such as the evaluation of the viral DNA load, virus isolation, HLA characterization of the host and IgG isotype analysis. These investigations are feasible and they will be part of our next investigation.

## Materials and Methods

### Human Samples

A total of 591 serum samples were collected at the University Hospital of Ferrara, Department of Neurology and Clinical Laboratory Analysis. Human sera were from discarded clinical laboratory analysis samples, anonymously collected, coded with indications of age, gender and pathology, if any. Sera from MS patients (n = 93), as well as from other inflammatory neurologic (OIND) (n = 77), and non-inflammatory neurologic diseases (NIND) (n = 81) affected patients and healthy subjects (HS) with a different mean age, HS1, HS2 (n = 180, mean age  = 40 yrs; n = 160, mean age  = 51 yrs, respectively) were analyzed for IgG antibodies reacting to SV40 viral capsid protein (VP) mimotopes, represented by synthetic peptides, which mimic VP antigens (Table 1). To this purpose indirect ELISA was set up employing synthetic peptides, which correspond to SV40 VP 1-2-3 mimotopes, together with an unrelated human peptide, hNPS, employed as a negative control of the serum reactivity [34]. Informed written consent was obtained by patients/individuals. The project was approved by the County Ethical Committee, Ferrara, Italy.

### Synthetic Peptides

Computer assisted analyses allowed us to select 2 specific SV40 peptides, from the late viral region by comparing the three capsid proteins, VP 1-2-3 from SV40, with the amino acids of the human BK (BKV) and JC (JCV) polyomaviruses which are highly homologous to SV40, as well as with other, less homologous polyomaviruses [20], [22]. Previous ELISA results indicated that the two SV40 peptides did not cross-react with the BKV and JCV hyperimmune sera that were employed as controls [20], [22]. The two peptides belong to the VP1/VP2/VP3 viral capsid proteins (VP1 ID: 1489598); VP3 ID: 9486895; web site, http://www.ncbi.nlm.nih.gov/nuccore). The amino acid sequences of the two peptides, known as VP1 B and VP2/3 C, respectively, are as follows:

VP1 B: NH2- NPDEHQKGLSKSLAAEKQFTDDSP- COOH

VP2/3 C: NH2- IQNDIPRLTSQELERRTQRYLRD- COOH

VP1 B and VP2/3 C mimotopes were selected as they react specifically in indirect ELISA with the rabbit hyperimmune serum that had been experimentally immunized with SV40 (positive control serum). SV40-positive and SV40-negative human sera were also employed as controls. These SV40 control sera, selected by the neutralization assay, were from our collections [19], [20], [21], [22] (see below the other technical details). BKV and JCV hyperimmune rabbit sera did not react with VP1 B or VP2/3 C peptides (negative control sera). The amino acid residues of the two specific SV40 VP peptides show low homology with the BKV, JCV and other polyomaviruses VPs [20], [22]. The characterization of these two peptides were published before [20], [22]. The synthetic peptides were synthesized using standard procedures and were purchased from UFPeptides s.r.l., Ferrara, Italy [20].

### Indirect Enzyme-Linked Immunosorbent Assay (ELISA)

Indirect ELISA was developed and standardized to detect specific antibodies against SV40 in human sera using VP1 B VP 2/3 C synthetic peptides [20], [22]. *Peptide coating*. Plates were coated with 5 µg of the selected peptide for each well and diluted in 100 µl of Coating Buffer, pH 9.6 (Candor Bioscience, Germany). *Peptide blocking*. Blocking was made with 200 µl/well of the Blocking Solution, containing the casein (Candor Bioscience, Germany) at 37°C for 90 min. *Primary antibody adding*. Different wells were covered with 100 µl containing the following sera: positive-control, represented by immune rabbit serum containing anti-SV40 antibodies, negative controls represented by immune sera anti-BKV and anti-JCV, and three human serum samples, which were found to be SV40 negative in our previous investigations [20], [22]. Sera under analysis were diluted at 1∶20 in Low Cross-Buffer, pH 7.2 (Candor Bioscience, Germany). *Secondary antibody adding*. The solution contained a goat anti-human IgG heavy and light chain specific peroxidase-conjugate (Calbiochem-Merck, Germany) diluted 1∶10,000 in Low Cross-Buffer. *Dye treatment and spectrophotometric reading*. Samples were treated with 100 µl of 2,2'-azino-bis 3-ethylbenzthiazoline-6-sulfonic acid (ABTS) solution (Sigma-Aldrich, Milan) and then read on the spectrophotometer (Thermo Electron Corporation, model Multiskan EX, Finland) at a wavelength (λ) of 405 nm. This approach detects the color intensity in wells where the immunocomplexes were formed by optical density (OD). *Cut-off determination*. The cut-off point was determined in each assay by an OD reading of three negative controls, that were added to the standard deviation and multiplied three times (+3SD). The choice of using the mean plus 3 times the standard deviation is to eliminate false positive samples and to increase the specificity of the ELISA test

Sera with antibodies against SV40 were considered VP-positive upon reacting to both peptides of the late region and when sera that had been analyzed three times, with independent experiments, by indirect ELISA testing gave the same positive result.

### SV40 specificity of the indirect ELISA employing synthetic peptides which mimic the VPs antigens

In previous investigations, comparative computer assisted analyses by BLAST program were carried out with the SV40 VP peptides B and C and the corresponding amino acid (a.a.) sequences of the new human polyomaviruses (HPyV) and hundreds of different BKV and JCV serotypes [20], [22]. Results indicate a low homology for the BKV and JCV prototypes and other polyomaviruses. [20], [22]. Indirect ELISA data indicate that the two SV40 peptides B and C did not cross-react with the BKV and JCV hyperimmune sera (negative controls), as described before [20], [22]. Briefly, hyperimmune sera against SV40 and BKV were obtained in rabbits that had been inoculated with purified viral stocks as previously reported [20]. The serum against JCV was kindly provided by Dr. Major, NIH, Bethesda (MD), U.S.A [20], [22]. The immune serum anti-BKV was titered using a hemagglutination inhibition (H.A.I.) test employing human erythrocytes group 0, Rh^+^. Anti SV40 serum was titered by neutralization assay [20].

SV40 VP1 B and VP2/3 C mimotopes were selected as they react specifically in indirect ELISA with the rabbit hyperimmune serum that had been experimentally immunized with SV40 (positive control) [20], [22], and with SV40-positive human sera. These SV40-positive human sera, from our collection, were analyzed before by SV40 neutralization assay [19], [20]. The human peptide hNPS, a.a. sequence SFRNGVGTGMKKTSFQRAKS [34] was employed as a negative control peptide [20], [22].

Serum samples tested by indirect ELISA diluted at 1/20 were considered SV40-positive when above OD = 0.17–0.19, according to the spectrophotometric reading. Indeed, this cut-off point represents the value that discriminates SV40-negative (sample with OD below 0.17–0.19) from SV40-positive samples (OD above 0.17–0.19). The positive controls, represented by the SV40 hyperimmune rabbit serum, had an OD of up to 1.8, while the two JCV and BKV hyperimmune rabbit sera, which were employed as negative controls, had an OD of less than 0.1. In each ELISA plate 3 SV40-positive and 3 SV40-negative human sera, from our collections [19], [20], [21], [22] were also added. The selection of these human sera was based on their SV40-neutralization activity [19], [20].

### Statistical analyses

All analyses were performed by Prism 4.0 (GraphPad software). For all tests, we considered *P* values < 0.05 to be statistically significant. To determine significances between two groups we used two-sided chi-square test. The serologic profile of serum antibody reactivity to SV40 mimotopes was statistically analyzed using Anova and Newman-Keuls Comparison test.
